# Research Insights on Neural Effects of Auditory Deprivation and Restoration in Unilateral Hearing Loss: A Systematic Review

**DOI:** 10.3390/jcm9030812

**Published:** 2020-03-17

**Authors:** Jolijn Vanderauwera, Elisabeth Hellemans, Nicolas Verhaert

**Affiliations:** 1 Psychological Sciences Research Institute, Université Catholique de Louvain, 1348 Louvain-la-Neuve, Belgium; jolijn.vanderauwera@uclouvain.be; 2 Institute of Neuroscience, Université Catholique de Louvain, 1348 Louvain-la-Neuve, Belgium; 3 Department of Otolaryngology, Head and Neck Surgery, University Hospitals Leuven, 3000 Leuven, Belgium; elisabeth.hellemans@student.kuleuven.be; 4 KU Leuven, Research Group ExpORL, Department of Neurosciences, 3000 Leuven, Belgium

**Keywords:** brain plasticity, cochlear implant (CI), congenital deafness, cross-modal reorganization, intra-modal reorganization, postlingual deafness, neuroimaging, single-sided deafness, unilateral hearing loss

## Abstract

Neuroplasticity following bilateral deafness and auditory restoration has been repeatedly investigated. In clinical practice, however, a significant number of patients present a severe-to-profound unilateral hearing loss (UHL). To date, less is known about the neuroplasticity following monaural hearing deprivation and auditory input restoration. This article provides an overview of the current research insights on the impact of UHL on the brain and the effect of auditory input restoration with a cochlear implant (CI). An exhaustive systematic review of the literature was performed selecting 38 studies that apply different neural analyses techniques. The main results show that the hearing ear becomes functionally dominant after monaural deprivation, reshaping the lateralization of the neural network for auditory processing, a process that can be considered to influence auditory restoration. Furthermore, animal models predict that the onset time of UHL impacts auditory restoration. Hence, the results seem to advocate for early restoration of UHL, although further research is required to disambiguate the effects of duration and onset of UHL on auditory restoration and on structural neuroplasticity following UHL deprivation and restoration. Ongoing developments on CI devices compatible with Magnetic Resonance Imaging (MRI) examinations will provide a unique opportunity to investigate structural and functional neuroplasticity following CI restoration more directly.

## 1. Introduction

Over the past decades, knowledge on neuroplasticity following sensory deprivation and its underlying mechanisms has rapidly expanded, also in the domain of the central auditory system. Sensory deprivation of the bilateral hearing system in patients is referred to as (bilateral) deafness. Structural cerebral changes, and functional activity changes following deafness have been frequently investigated with the majority of the research focusing on congenital/prelingual bilateral deafness. In addition to the population of patients with congenital and acquired bilateral deafness, in clinical practice, a significant number of patients present severe-to-profound unilateral hearing loss (UHL). UHL is defined as an asymmetric hearing loss with a normal hearing on the opposite side, also referred to as single-sided deafness (SSD) and occurs in around 1 out of 1000 children in its congenital form [1]. The review by Van Wieringen et al. on the etiology of congenital UHL reported that congenital causes mainly consist of cytomegalovirus infection, congenital nerve deficiency, syndromal causes, bacterial meningitis, inner ear malformation, or unknown etiology. In older children and adults, predominant acquired causes of UHL include vestibular schwannoma (34 per million per year), trauma (due to temporal bone fracture), Ramsay Hunt syndrome (herpes zoster virus), auto-immune hearing loss, Menière’s disease and idiopathic sudden sensorineural hearing loss (incidence of 27 per 100,000), chronic otitis media, and failed middle ear surgery, rendering the prevalence much higher [1,2]. UHL has been shown to decrease sound localization, a central binaural hearing process [3]. Moreover, UHL during early childhood negatively impacts the development of oral language (for a review on language impact, see Van Wieringen et al. [1]). Compared to bilateral deafness, dedicated research on UHL is more recent and less is known about the neural changes following UHL. Hence, the primary objective of the present study is to summarize and evaluate current research insights on structural and functional brain plasticity following unilateral hearing deprivation caused by UHL. In the present systematic review, neural plasticity is studied both in auditory and non-auditory cortices. We thereby especially emphasize factors that can potentially influence the observed neural plasticity, such as the age of onset of the UHL, the duration of the UHL, and the deafness side. For the age of onset, we seek to disambiguate structural and functional plasticity findings for UHL deprivation that occurred during child development (congenital and/or prelingual) and in the elderly (postlingual), as the mechanisms and patterns of cortical reorganization in both populations may differ [4]. Given the ageing population in most developed countries, there is an increase in the prevalence of acquired age-related hearing loss, emphasizing the need for insights in the neural plasticity specific to this population. Postlingual hearing loss is indeed the third most prevalent chronic medical condition amongst elderly patients after arthritis and hypertension [5]. Furthermore, we aim at evaluating the effect of side of deafness on the neural plasticity following UHL as the auditory cortex is known to display lateralization asymmetries even in the case of bilateral lateralization. Hence, studying the case of unilateral auditory deprivation will enhance our knowledge on the functioning of the entire auditory system.

Besides the emerging investigation of sensory deprivation in the past decades, medical innovations have made it possible to investigate the effects of sensory restoration in the auditory domain. Mild and moderate sensorineural hearing loss is often managed with digital hearing aids, while conductive hearing loss can be treated with middle ear surgery or a bone conduction device. A combination of both conductive and sensorineural, named mixed hearing loss, can be treated with surgery or with the implantation of an acoustic hearing implant [6]. Cochlear implants (CI) provide hearing in deaf subjects, by transducing auditory stimuli as electrical signals to the cochlear nerve. Up until the recent past, UHL remained untreated or a contralateral routing of signals was proposed with a hearing aid or bone conduction implant [7]. There is, however, a clinical demand for bilateral auditory restoration in this very communicative world. Although valuable, the solution of contralateral routing only stimulates the hearing ear, leaving the deafened ear unstimulated. In more recent years, CIs are becoming a viable option for the deafened side, restoring bilateral input, i.e., two ears separately, to the auditory system. Although evidence is sparse, there seems to be a window of opportunity for CI to maximize performance of the child and this window has been set to 36 months after birth [1,8]. Less stringent data on time window availability of CI is available in adults, leading to clinical opportunism and the absence of guidelines. Applying CI in patients with UHL has also been demonstrated to increase sound localization after six months of hearing through the CI [3]. Hence, current CI in UHL might support binaural hearing processes. Nevertheless, longer duration of hearing deprivation in the deaf ear was suggested to decrease sound localization ability, a process that can be considered to be caused by further degeneration of the auditory pathway and/or limitations in neural plasticity after long-term deprivation. In addition to sound localization, other benefits have been reported for the use of CI in patients with UHL. Among these are positive impact on incapacitating tinnitus, i.e., ringing in the ears, with CI in case of acquired UHL [9] and increased perceived quality of life [10,11]. Notwithstanding clear benefits and despite increasing clinical evidence, in many countries, CIs are not yet reimbursed in adults and children with UHL [1]. One factor that might impact clinical application of CI in UHL and the lack of reimbursement can be considered the lack of firm knowledge about the factors contributing to successful hearing restoration. Considerations for evidence-based practices for patients with UHL cannot be drawn from the processes of auditory deprivation and restoration in patients with bilateral deafness. Hence, in addition to summarizing the impact of unilateral auditory deprivation on the brain, the second objective of this study is to provide a comprehensive overview of current research insights on the neural plasticity after unilateral hearing restoration by means of a CI. Given the current challenges of compatibility of CI devices with the large-gradient magnetic fields of an MRI scanner, we will implement research insights obtained by means of animal research. Again, we will especially emphasize factors that can potentially influence the observed neural plasticity, such as the age of onset of the UHL and the duration of the UHL. For the duration of UHL, we are especially interested in evaluating evidence on the potential influence of duration of the UHL on CI outcome. We conclude with an evaluation of the current insights, the research methods currently applied and a proposal for future neuroscientific research, in line with the goal of the research field to gain insight on the large inter-individual variability following CI, eventually leading to an updated definition of appropriate candidates with UHL for cochlear implantation.

## 2. Materials and Methods

### 2.1. Design and Study Sample

**Figure 1:**
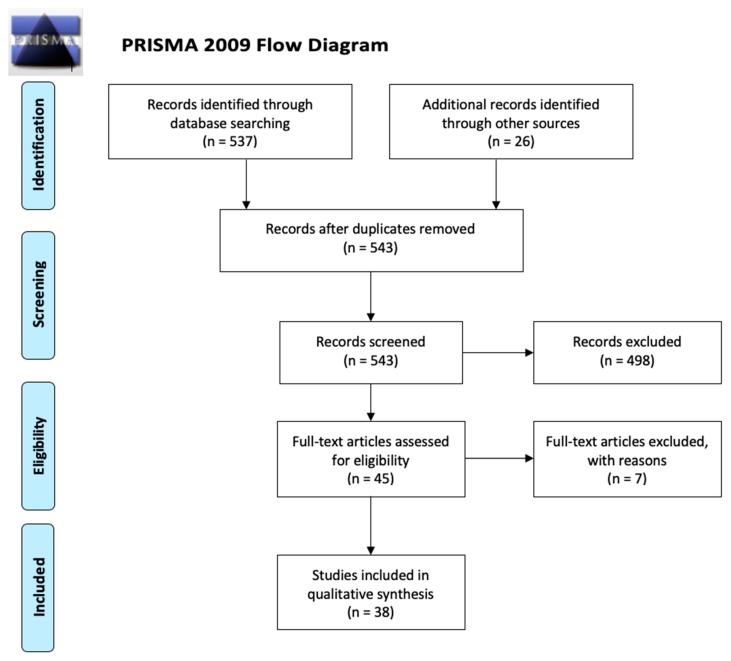
Overview of selection of relevant records according to the PRISMA guidelines (2009).

Papers were selected according to the Preferred Reporting Items for Systematic Reviews and Meta-Analyses (PRISMA) flow diagram [12] guidelines (see Figure 1). The database search was carried out in MEDLINE (PubMed) in February 26 2020 using medical subject headings (MeSH-terms) and free text terms. The purpose of the database search was to identify studies published between 2000 and 2020 that either investigated the brain correlates (structural and functional) of unilateral hearing deprivation caused by UHL or SSD and on the other hand, the restoration of UHL by means of CI. Given the current challenges of conducting neuroimaging research in patients with CIs, animal studies were allowed for the investigation of the UHL restoration effects.

The following MeSH-terms and free text terms were applied:

((“hearing loss, unilateral”[MeSH Terms]) OR (“unilateral hearing loss”[All Fields]) OR (“hearing”[All Fields] AND “loss”[All Fields] AND “unilateral”[All Fields]) OR (“unilateral hearing loss”[All Fields]) OR (“unilateral”[All Fields] AND “hearing”[All Fields] AND “loss”[All Fields]) OR (“single-sided deafness “[All Fields]) OR (“single”[All Fields] AND “sided”[All Fields] AND “deafness”[MeSH Terms])) AND ((“neuroimaging” [MeSH Terms]) OR (“functional MRI”[All Fields]) OR (“structural MRI”[All Fields]) OR (“diffusion weighted imaging”[All Fields]) OR (“Diffusion Tensor Imaging”[MeSH Terms]) OR (“cochlear implant”[MeSH Terms]) OR (“cochlear implantation”[MeSH Terms])) AND (“2000”[PDAT]: “3000”[PDAT]).

The database search resulted in 532 records. In addition, references to magnetic resonance imaging (MRI) studies on participants with UHL were adopted from the paper by Heggdal and colleagues [13], who performed a peer-reviewed search in March 2015. Furthermore, Embase and Google Scholar were consulted for detecting relevant studies on patients with UHL and CI patients, adopting both MRI or electroencephalography (EEG) protocols. Hence, 26 additional records were identified through searching other sources (see Figure 1).

After duplicate removal in Zotero (Zotero, 2017), titles and abstracts of 543 records were screened for eligibility using Rayyan [14], an online application for screening of study records. Exclusion criteria were applied to rule out irrelevant articles. The first pertinent exclusion criterion was labelled, although in many cases, more than one criterium applied. Studies that reported on patients with bilateral hearing loss (*n* = 49) and/or bilateral cochlear implantation (*n* = 72) were excluded for further analyses. Meta-analyses and reviews were excluded (*n* = 58). Studies that did not report on neuroimaging measures (MRI and/or EEG) were excluded (*n* = 242). Studies with a primary focus on tinnitus were excluded (*n* = 32), as well as studies written in another language than English (*n* = 8). Finally, studies that were not relevant or specific enough for the subject matter were excluded (*n* = 37). Hence, 498 records were excluded after screening and 45 full-text articles were all assessed for eligibility by two raters (J.V. and N.V.). Out of these 45 articles, seven were excluded. Exclusion reasons were a primary focus on the acute impact of sudden UHL (*n* = 4), pilot studies (*n* = 2), and animal studies that merely investigated the neural effects of auditory deprivation without investigating the neural effects of auditory restoration (*n* = 1). Hence, 38 articles were included for further analysis. Please see Figure 1 for an overview of the different steps taken to asses for eligibility according to the Prisma guidelines (Prisma Flow diagram, 2009). An overview of the included articles is presented in Table 1 for the studies on the neural effects of unilateral auditory deprivation (i.e., research objective 1) and in Table 2 for the studies investigating the effect of unilateral auditory restoration (i.e., research objective 2).

### 2.2. Neural Analyses Methods

The studies evaluating the effect of UHL on the brain included in the present study either used MRI or EEG to investigate neural changes following UHL. MRI was adopted to investigate functional and structural changes with a high anatomical specificity. Given that the majority of currently used CIs (during the reported studies) are not compatible with the large magnetic field induced by MRI and provide significant metallic artefacts, this technique was exclusively used in non-CI UHL patients. Functional MRI (fMRI) allows for indirect measures of neuronal activity by means of blood oxygenation level dependent (BOLD) measurements and is the neuroimaging technique adopted by the majority of the studies evaluating the neural effects of UHL (see Table 1 and Table 2). To investigate anatomical brain changes, T1/T2 MRI scan can be used to evaluate morphological changes in the brain that can be related to hearing loss. These neuroimaging scans are frequently applied for clinical purposes, for example, for the radiological evaluation of anatomical malformations that might explain the origin of a measured hearing loss. In addition, anatomical T1/T2 MRI scans are used for research purposes, most frequently to compare the anatomical gray matter properties of a group of individuals with hearing loss relative to a group of normal hearing controls. The thickness, surface area, and volume of a specific gray matter volume can be studied between groups by applying this approach. Apart from investigating structural properties of specific brain regions, diffusion-weighted imaging (DW-MRI) can be applied to measure structural white matter properties in regions and brain connections. DW-MRI is a structural imaging technique that measures the diffusion of water molecules, which will diffuse faster parallel (axial) than perpendicular (radial) to the trajectory of white matter tissue (for more information see [15,16]). By applying large magnetic field gradients in distinct directions, this phenomenon, known as anisotropy, can be studied using different models. The first and most frequently applied model is the diffusion tensor imaging (DTI) model [17]. For quantitative analyses, multiple indices can be used to derive indirect information on structural white matter organization, such as mean diffusivity (MD), axial diffusivity, radial diffusivity, and fractional anisotropy (FA). FA measures the degree to which the diffusion of water molecules is anisotropic and is the most widely applied index [15]. FA values range between 0, i.e., completely isotropic and 1, i.e., completely anisotropic. Finally, EEG provides a measure of brain function that was used to investigate functional changes in response to stimuli. EEG uses the principle of differential amplification, or recording voltage differences between different points using a pair of electrodes that compares one active exploring electrode site with another neighboring or distant reference electrode. By this, EEG waveforms are generated and we can measure brain activity [18].

## 3. Results

### 3.1. Functional and Structural Brain Plasticity in UHL

Brain plasticity related to monaural hearing is reviewed in this section. In the first and second section, respectively, functional and structural brain plasticity is evaluated with special emphasis on factors that can explain different findings between studies such as age of onset of the UHL, disentangling UHL that occurred during child development and in the elderly, and the duration of the UHL. In the final section, evidence on the effect of the side of the UHL on neural plasticity is reviewed.

#### 3.1.1. Functional Plasticity

Functional MRI

fMRI studies show that deafness affects the auditory cortical areas. It is known that in normal hearing (NH) subjects, the auditory cortex displays lateralization asymmetries despite binaural input [19]. For example, in NH persons, monaural auditory stimulation leads to greater contralateral hemisphere activation (magnitude of evoked BOLD responses) versus ipsilateral activation [20]. Studies of patients with UHL report changes in lateralization of the auditory cortex. For example, the study by Bilecen et al. [21] reported on a 53-year-old individual with sudden UHL after cochlear nerve resection for schwannoma surgery. In this study, hemispheric responses were measured using fMRI prior to and repeatedly after the occurrence of UHL. Interestingly, while the contralateral responses were confirmed prior to the occurrence of UHL, the patient showed compensatory reorganization that progressed over the course of around one year, resulting in stronger bilateral responses to stimulation of the intact ear. This type of increased ipsilateral response was also reported by Van der Haegen et al. [22]. In fact, the authors investigated a sample of seven adults with right UHL and investigated whether monaural deprivation of the right ear would affect language lateralization, classically towards the left hemisphere. No atypical language lateralization patterns were observed, which is in line with higher ipsilateral responses as a result of monaural hearing through the left ear. The study by Burton et al. [20] further seems to point towards the idea that this type of UHL induced cortical reorganization in the auditory cortex is not specific to patients who can be considered unilateral deaf, but can also be observed in less than profound UHL. Note that currently, evidence is lacking on this type of functional reorganization in children with UHL.

In addition to studies investigating the effect of auditory stimulation in patients with UHL, other studies evaluated the effect of UHL on multi-model integration and the default mode network (DMN). For example, the study by Schmithorst et al. [23] reported on a group of children aged 7–12 years old (*n* = 21) with UHL, with a general duration over 2 years. They revealed that children with severe to profound UHL displayed decreased activation in right hemispheric regions of the visual processing pathway, suggesting differences in cross-modal integration. In addition, children with UHL showed decreased deactivation of regions of the DMN. This type of functional reorganization in regions that serve a role in multi-model integration and the DMN was confirmed by the study of Wang et al. [24], who applied resting-state fMRI in a group of 34 adults with UHL and a group of 22 normal hearing controls, and by the study of Zhang et al. [25], who used a resting-state connectome analysis in 21 adults with UHL compared to 21 matched NH controls (see also Zhang et al.) [26]. The latter study also reported that changes in intra-network connections were related to the duration of UHL, suggesting the importance of early restoration. In addition, the resting-state fMRI studies by Tibbetts et al. [27] and Jung et al. [28] showed that a group of children with UHL, aged 7–17 years, presented differences in many different brain network connections involved in executive functioning, memory formation, visual processing, cognition and language comprehension, in addition to differences in the auditory processing regions. Again, similar findings were reported in a group of adults with UHL, relative to a group of normal hearing controls [29].

Only a handful of studies evaluated the effect of the ear of UHL on cortical reorganization, and the large majority of them applied functional MRI measures. One of the first studies investigating the neural differences between left and right UHL was the study by Schmithorst et al. [30]. This study indeed showed preliminary evidence for differences between neural networks supporting auditory processing in four patients with left and four patients with right UHL. This evidence was confirmed by later research. The study by Burton et al. [31], for example, evaluated the effect of side of UHL in a group of 26 adults with largely varying duration of UHL, relative to 24 normal hearing controls. Therefore, the authors adopted an fMRI paradigm to investigate activation magnitudes of the BOLD signal in core, belt, and parabelt regions of the auditory cortex. They revealed that side of UHL indeed affected BOLD responses. More specifically, right ear stimulation lead to more bilateral activation of core and belt auditory regions in patients with UHL, pointing towards the plasticity of the right hemispherical auditory regions. Left ear stimulation, on the contrary, only resulted in larger ipsilateral activation in the posterior auditory cortex. The authors propose that the left hemispherical auditory regions were more resilient to reduced auditory stimulation from the deaf right ear than the right hemispherical counterparts. Similar findings for right ear stimulation were found by Hanss et al. [32] in a group of 18 post-lingual UHL patients, although no effect of left ear stimulation was reported in this study. The study by Yamamoto et al. [33] evaluated effect of side of deafness on speech in noise perception in adults with UHL using fMRI. They revealed that patients with right UHL showed altered brain activity in right posterior auditory regions, while no alterations were found for patients with left UHL. The resting-state connectivity study by Zhang et al. [34] further analyzed the effect of left vs. right UHL on cross-modal connectivity patterns in a sample of patients ranging from 15 to 70 years old with acquired UHL for 2 to 50 years. The results of this study revealed that patients with left UHL (right ear stimulation) had stronger cross-modal connectivity between the (non-deprived) left auditory cortex and regions of the visual and sensorimotor networks relative to normal hearing individuals. No such difference was found for patients with right UHL (left ear stimulation). The study by Zhang et al. [35] revealed that adults with long-term left UHL presented changes in the DMN, while again no such changes were present in patients with right UHL. The studies by Zhang et al. [26,36] further pointed to left and right UHL differences in the visual network, DMN, and subcortical regions, with both alterations specific to patients with left UHL and alterations specific to patients with right UHL. These findings again point towards a different neural signature for left and right auditory deprivation, and these differences extend the primary regions for auditory processing.

Besides the studies mentioned above in adult populations, the study by Propst et al. [37] evaluated the effect of the ear of UHL in a group of 6 children with left UHL and 6 children with right UHL. First, their results indicated that children with UHL had less overall activation of auditory regions. In addition, right ear stimulation lead to more activation of the visual association areas compared to left ear stimulation while left ear stimulation lead to failure in activation of attention areas.

Electroencephalography

In addition to fMRI, EEG was used to investigate the neural effects of monaural hearing deprivation. In a similar vein to fMRI results, reduced hemispheric asymmetries were also reported by Maslin et al. [38,39], applying EEG measures in patients (age 40–75 years) to evaluate cortical reorganization within and after the first 6 months of UHL following acoustic neuroma removal. Another study by Pross et al. [40] applied MEG in adults with long-term UHL and confirmed hemispherical alterations in response to sound. More specifically, patients with UHL showed a decreased interhemispheric latency. With respect to the effect of side of UHL, the EEG study by Khosla et al. [41] reported on similar findings as reported by the fMRI study by Hanss et al. [32], i.e., reduced inter-hemispheric amplitude differences recorded with auditory evoked potentials following right ear stimulation, in the absence of an effect in left ear stimulation.

#### 3.1.2. Structural Plasticity

Anatomical T1/T2 neuroimaging

A study by Lipschitz et al. [42] examined structural neuroimaging scans (CT and MRI) of 170 pediatric patients (age 0–18 years) with UHL. They reported that in nearly half of the sample, structural malformations were detected that could be associated with the hearing loss. More specifically, 51% had a cochlear nerve deficiency, 40% showed cochlear dysplasia, and 27% had an enlarged vestibular aqueduct. Hence, the use of neuroimaging techniques aid in the evaluation of pediatric UHL. In a similar vein, Clemmens et al. [43] investigated the prevalence of cochlear nerve deficiency in 128 children aged between 3 weeks–16 years. They reported on a prevalence of 48% of cochlear nerve deficiency in children with severe to profound UHL, and even 100% in infants with congenital UHL. Hence, cochlear nerve deficiency can be seen as a common cause of UHL, especially in severe to profound congenital UHL.

In an important addition to investigating morphological malformations that can explain the observed UHL, T1/T2 weighted volumetric MRI images can be used to study gray matter and white matter deviances in specific regions. The study by Yang et al. [44] applied voxel-based morphometry (VBM) to evaluate volumetric changes in a group of 14 patients (age 41–60 years old) with right UHL and a large variation in duration of the hearing loss. The results indeed seem to confirm that patients with UHL have decrease gray and white matter volume in a broad set of auditory and non-auditory cortices, consisting of bilateral, and unilateral regions such as the bilateral posterior cingulate gyrus, precuneus, left superior-, middle-, and inferior temporal gyrus, right parahippocampal gyrus, and lingual gyrus. In addition to auditory processing, these regions support functions such as visual and spatial processing, language, semantic and episodic memory, and some of the regions are part of the DMN.

Diffusion Tensor Imaging (DTI)

Auditory deprivation may affect the structural properties of white matter regions and connections. DTI studies by Lin et al. [45] and Wu et al. [46] investigated the properties of two of these regions that are located along the auditory pathway, i.e., the Lateral Lemniscus (LL) and the Inferior Colliculus (IC) applying the DTI model. The study by Lin et al. [45] compared a group of individuals with bilateral HL (*n* = 15), a group of individuals with UHL (*n* = 12) and a group of normal hearing controls (*n* = 10). The mean age of the patients was 32 years old (SD = 12 years) with a large duration range of the HL, i.e., 1 to 48 years. Lin and colleagues reported that the groups of individuals with HL (both bilateral and unilateral) showed reduced FA values in both the LL and IC compared to the normal hearing controls. In addition, for the UHL group no differences were reported between the findings within the hemispheres contralateral and ipsilateral to the deaf ear. The study by Wu et al. [46], on the other hand, who reported on the same regions in a sample of 19 patients with a broad age range (8–60 years old) and long-term UHL, did report on a hemispherical difference in the FA values of the LL and IC regions, with respect to the deaf ear. More specifically, a decrease in FA was observed in the LL and IC on the contralateral side of the deaf ear, in the absence of such decrease on the ipsilateral side. The pilot study by Rachakonda et al. [47] revealed decreased FA in the left LL in a group of children with UHL, irrespective of side of deafness. Another study by Vos et al. [48] applied DTI to investigate FA and MD in the auditory nerve, a pathway that is particularly hard to reconstruct in vivo given that it is a very small structure. They compared a group of five patients with UHL (36–64 years old) who had a long-term profound UHL and revealed decreased FA in bilateral auditory nerves relative to the normal hearing individuals. Hence, although evidence points towards some alteration in white matter properties along the auditory pathway, the impact of age of onset and duration of the hearing loss of the UHL remains largely to be determined.

### 3.2. Auditory Restoration by Cochlear Implantation

Cochlear implants (CI) provide hearing in deaf subjects, by transducing auditory stimuli as electrical signals to the cochlear nerve. Despite increasing evidence, in many countries, CIs are not yet reimbursed in adults and children with UHL [1]. There is, in general, a large variability in CI outcome regarding speech perception and language development. Important influencing factors, yet highly correlated, are age at implantation, duration of deafness, age of onset of deafness, and extent of experience with CI [30,31]. Researchers proposed that cortical structural and functional changes, induced by hearing deprivation and restoration, explain additional variability and inter-individual variation [49]. A study by Kral et al. [50] indeed confirmed that the outcome of auditory restoration in the case of monaural stimulation was linked to the developmental timing of hearing restoration. They showed that in congenital deaf cats, early restoration of monaural hearing (i.e., during the peak of functional cortical synaptogenesis) by means of a single CI lead to massive reorganization of aural preference, favoring the hearing ear. This effect disappeared when monaural hearing was restored later in development. Hence, in the case of early UHL in humans, the hearing ear can be considered to become functionally dominant. This effect was confirmed in rats with congenital UHL, showing a bilateral activation of neurons in the cochlear nucleus [51]. Functional dominance refers to the process where the cortex ipsilateral to the hearing ear demonstrates a functional shift from the sensory deprived ear towards the hearing ear [50]. The same mechanism can be considered to explain the inferior outcome of CI in a second implanted ear in case of bilateral deafness [52]. These results were confirmed by another study applying an animal model to compare the impact of bilateral hearing loss vs. UHL by means of binaural and monaural stimulation applying CI in congenital (bilateral or unilateral) deaf cats [50]. UHL did not result in reduced cortical responsiveness to sound, in contrast to bilateral hearing loss, but there was a clear indication for hemisphere-specific reorganization and functional dominance. Functional dominance of one ear can thus be considered to decrease the neural plasticity following restoration of the deaf ear and therefore impact the clinical outcome of CI in congenital UHL [50]. In addition, the study by Basta et al. [53] in guinea pigs with induced UHL in adulthood showed that CI implantation reduced the spontaneous activity that is usually elevated upon deafferentiation. Hence, CI in the case of UHL can facilitate the homeostasis of the auditory network. These results indicate that binaural hearing should be restored early in development, emphasizing the importance of early CI in the case of congenital UHL. A side note to these findings is that, although early implantation seems beneficiary to avoid neurofunctional dominance of the hearing ear, the study by Firszt et al. [54] reported on a case study of a patient who experienced 40 years of congenital, yet conductive, UHL prior to (non-CI) surgical auditory restoration. Interestingly, fMRI results indicated that hearing restoration after long-term UHL resulted in increased contralateral auditory cortex responses, as well as improvements in speech perception and sound localization. Hence, in this case, neural plasticity was observed even after 40 years of congenital UHL, although it has to be considered that bone conduction might have played a role in this single case with stapes footplate fixation. Hence, despite the neural disadvantage of the long-term deaf ear, activation of the auditory cortex still seems possible, by means of appropriate training [50]. In most recent years, the first evidence was reported on the neural effects of CI implantation. Two independent case studies applied cortical auditory evoked potentials (CAEPs) to measure the neural changes post-CI. One case was a child with right UHL from birth who received a CI at the age of 8 [55], while the other case was a child with progressive idiopathic right UHL beginning at the age of 5, who received a CI at the age of 9 [56]. Although the precise neural effects of the monaural restoration are hard to interpret in the developmental population by means of case studies, both studies revealed a high degree of neuroplasticity in the auditory cortex, evidenced at the age of 7–8 years with a large variability in duration of hearing loss. Furthermore, clinical improvements were apparent, as both children significantly improved post-CI with respect to speech recognition and sound localization abilities and their performance was within the typical range. The study by Polonenko et al. [57] investigated five children with congenital left UHL, treated with a CI. The EEG study measured the impact of CI implantation in the acute, early chronic, and chronic stages post-CI. Interestingly, while in the acute post-CI stage, the responses from stimulation of the implanted ear were measured predominantly in the ipsilateral hemisphere; this atypical pattern of responses was resolved 12 months post implantation. Hence, in children with congenital UHL, early implantation can rapidly restore neural responses to bilateral auditory input, needed for binaural processes such as sound localization. Finally, one larger sample study on 10 adults with restored UHL by means of a CI was conducted, comparing performance of the NH ear to the CI ear [58]. They showed prolonged N2/N4 latency responses for word classification for stimulation of the CI ear, compared to the NH ear. These results indicate hampered auditory-cognitive integration in CI users and might explain behavioral variation in speech discrimination. The authors stress the importance of cognition for speech perception under adverse listening condition.

Finally, while initially, CI as a treatment option in UHL was only considered in cases of debilitating [59]; current indication criteria have been extended to UHL without tinnitus. Tinnitus is a common symptom of hearing loss that is especially difficult to treat. Several clinical studies on CI in UHL have reported an improved speech intelligibility in challenging listening situations, higher sound localization accuracy, and/or a decrease in tinnitus severity [60].

## 4. Discussion

In this review, we provided a comprehensive overview of studies investigating the impact of auditory deprivation in severe-to-profound congenital and acquired UHL, and the effect of sensory restoration by cochlear implants, on the cerebral and subcortical brain regions, by means of different research methods. We showed that in the past decade, important steps forward are taken in the research on the underlying neural plasticity of unilateral auditory deprivation and restoration. The main findings are 1) adults studies showed that functional brain changes following UHL resulted in a change in lateralization of processing auditory information, from predominant contralateral processing to a bilateral neural network; 2) evidence seems to point towards differences in neural plasticity with respect to the side of UHL; and 3) onset time of UHL impacts auditory restoration processes. However, research findings on the neural plasticity following unilateral auditory deprivation and restoration are still relatively scarce, and evidence is especially lacking on the structural neural changes following UHL. From a research perspective, it would have been more stringent to separate congenital and acquired UHL and their impact, but as separate neuroscientific evidence of both groups is indeed limited, we opted to review both groups together as insights on the neural plasticity will be intertwined and relevant for both groups. Changes in peripheral hearing impact upon cortical speech processing networks, with cascading effects for the neural processes supporting both perceptual and higher-level cognitive functions [1]. It is well known that alterations in neuroplasticity also occur after acquired UHL [38,39], however, those as a result of congenital deafness, thus including congenital UHL, pose severe challenges for the maturation of the brain [8,61]. Further research is required to allow for firm interpretation of the effects of duration of the hearing loss and onset of the UHL, two factors that hold the potential to be key predictors of restoration outcome by means of a CI, in case of normal cochlear anatomy and cochlear nerve presence.

A number of studies evaluated the functional and structural neural impact of UHL. The studies by Bilecen et al. [21] and Maslin et al. [38,39] applied an interesting approach to evaluate the trajectories of functional plasticity following the onset of UHL. The functional neural networks supporting auditory processing were evaluated with a longitudinal design, starting prior to the onset of UHL with follow-up within the first months after the onset of UHL (up to one year in the case study by Bilecen et al. [21]. They showed that UHL leads to more bilateral auditory networks, with a decreased contralateral processing pattern as seen prior to the onset of UHL. However, these studies were conducted in mixed populations with a large variability in duration of UHL and ranging from adulthood to elderly individuals. Hence, it remains to be determined how much age of the participants, age of onset, and duration of the UHL influence these functional plasticity findings. This is especially important when it comes to restoration of the UHL, which might be influenced by factors such as age and duration of the UHL with respect to its functional reversibility.

On the opposite site, studies investigating functional reorganization in multimodal integration processes and the DMN were conducted in children [19,21,38] as well as in adults with UHL [20,39]. This difference in populations might be driven by the fact that those studies primarily applied resting-state fMRI, an fMRI technique that does not require the participant to do a task in the scanner. We can conclude from these studies that unilateral auditory deprivation influences neural networks involved with a large variety of processes such as language comprehension, visual processing, executive functioning, memory etc. These functional alterations were observed both in the adult and the child population, however, further research is required to understand the trajectories of the observed functional alterations after the onset of UHL.

With respect to the structural gray matter changes in patients with UHL, there is only one study quantitatively evaluating volumetric changes in an adult population with a large variety of duration of UHL [30]. This study applied voxel-based morphometry (VBM), a measure evaluating cortical changes on a voxel-level that is not able to disentangle whether deviances are to be attributed to gray or white matter changes. Hence, evidence is largely lacking on gray matter changes in relation to UHL.

Interestingly, structural neuroimaging scans have been adopted to examine whether structural malformations could be detected that can explain the measured UHL [23,24]. In both studies, cochlear nerve deficiency was detected in 50% of the cases, and even 100% of the infants [43], showing that cochlear nerve deficiency is a common cause of UHL, especially in severe to profound congenital UHL. In addition to cochlear nerve deficiency, cochlear dysplasia and an enlarged vestibular aqueduct were detected as structural malformations which could explain the UHL [42]. Investigations of structural malformations are clinically relevant for the evaluation of the etiology of the hearing loss. Additionally, as this method is applicable in infants, it can aid the diagnostic process. While structural malformations are classically evaluated by the experienced human eye, developments in the field of computer sciences might in the future facilitate objective classification of the malformations. Indeed, in most recent years, a computer-aided diagnostic system has been developed for classification between left and right UHL in adult patients and hearing controls using T1 scans [62]. The application of this computer-aided diagnostic tool resulted in a correct classification in over 95% of the cases, thereby trespassing the accuracy of the experienced observer. To be of added value for clinical practice, further research is required to evaluate the application of this technique in younger developing populations, especially in infants. However, given that the technique should be considered as a ‘black box’, and that the results only contain a classification, without any qualitative information on the etiology, the application of it can never replace the clinical diagnoses by a trained individual, but can merely assist in the objectivation of the process.

Structural white matter changes have been studied along the course of the auditory pathway in a handful of studies applying the DTI model. The study by Wu et al. [46] showed that individuals with UHL had poorer structural organization, quantified by FA, in two regions of the auditory pathway on the contralateral hemisphere of the deaf ear, i.e., the lateral lemniscus (LL) and the inferior colliculus (IC). These findings are in line with the functional plasticity reported in adults with UHL (see above). However, the patients with UHL have a very wide age range, from 8 to 60 years old, and the results were not confirmed by another study with a broad age range as well as a large variety in age of onset [45]. Hence, although evidence points towards some alteration in white matter properties along the auditory pathway, the impact of age of onset and duration of the hearing loss of the UHL remains largely to be determined.

Furthermore, the studies investigating white matter organization of the LL and IC restricted the analyses to the evaluation within certain regions of interest (ROIs), although by means of DW-MRI one is capable of tracking structural connections between regions. One study applied the DTI model to bilaterally trace the auditory nerve and evaluate structural properties within the tract in patients relative to controls. FA in the auditory nerve appeared to be decreased bilaterally in five adult patients with long-term UHL. This study thus shows the potential for the reconstruction of small pathways. Nevertheless, the DTI model might not be most suited for this type of reconstruction of small structures. Moreover, although often interpreted as microstructural indices, the FA and MD indices of the DTI model do not allow to disambiguate microstructural from macrostructural properties. Applying higher-order models for analyzing DW-MRI, such as spherical deconvolution in combination with multi-shell multi-tissue scan acquisitions, hold the potential to enhance the reconstruction in this type of small structure pathways and tease apart microstructure from macrostructure [51].

Some studies specifically evaluated the effect of side of UHL on the neurofunctional plasticity, while no studies were found investigating this effect on structural plasticity. Overall, more alterations were seen as a result of left UHL than right UHL, both in the auditory regions and the DMN. Burton et al. [31] proposed that left hemispherical auditory regions were more resilient to reduced auditory stimulation from the deaf right ear than the right hemispherical counterparts. Moreover, a study in children with UHL showed that right ear stimulation lead to more activation of the visual association areas compared to left ear stimulation while left ear stimulation lead to failure in activation of attention areas. However, Yamamoto et al. [33] showed that patients with right UHL presented altered neural response to auditory perception in noise. Hence, differences in the neural effects of left and right UHL were evidenced, although clinically those populations are not differentiated. Future research would benefit from taking into account that evidence points towards differences with respect to the side of deafness, which can affect the behavioral outcome as well.

Finally, reviewing existing evidence on the ideal timing for restoration of the UHL resulted in interesting findings. First, the study by Kral et al. [52] investigated the effect of congenital UHL with monaural stimulation in cats. This study revealed that in the case of early congenital onset of UHL, the hearing ear became functionally dominant. Although this process can be considered advantageous in the case of monaural hearing, functional dominance of the hearing ear impacts neural plasticity following restoration of the deaf ear and therefore impacts clinical outcome. Hence, from a clinical perspective, early restoration of UHL can be considered advantageous for neural restoration. These results favor the early restoration of the UHL by means of a CI. Moreover, the results might explain the inferior outcome of implantations often seen at the ear that is implanted secondary, relative to the primary implanted ear. With respect to acquired UHL, the study by Bilecen et al. [21], investigated the impact of induced UHL due to cochlear nerve resection by means of fMRI. They showed that progressive compensatory processes, going from contralateral to bilateral brain responses in response to stimulation of the intact ear, took place along the course of approximately one year. Importantly, also in the case of acquired UHL, early restoration seems essential, at least within one year of the onset of UHL to avoid inferior CI outcomes. Overall, the findings of studies investigating the neural effects of hearing restoration by means of CI are in line with the idea of auditory critical periods, although these critical periods can potentially be reopened by means of auditory restoration in combination with focused training therapy [63,64]. So far, most studies have focused on the beneficial effect of adding a second ear, in terms of spatial and binaural hearing, i.e., what is the added benefit of a second CI-implanted ear. Recently, a longitudinal study on 6 UHL infants with early CI intervention was reported assessing linguistic and cognitive outcomes of children with congenital UHL in comparison to a non-implanted sample [65]. The children received a CI under the age of 2 years old and showed a beneficial effect in most children, in line with normal-hearing children with regard to linguistic skills and cognitive milestones. A larger sample is currently in a longitudinal follow-up. Similarly, more data are becoming available on CI treatment in adults with UHL.

**Figure 2:**
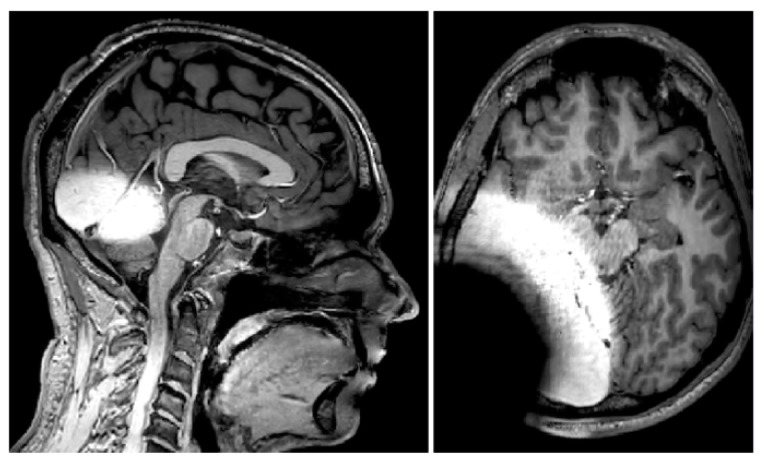
Visualization of the MRI artefacts caused by a unilateral cochlear implant (CI). In the left panel, the midsagittal plane has been visualized, showing large artefacts around the temporal lobe, while the inter-hemispherical corpus callosum is not hampered. The right panel shows an axial slice with extensive MRI artefacts.

Finally, to be able to identify functional network architecture differences in the brains of children and adults with UHL treated with cochlear implantation, MRI compatible protocols will need to be developed. Equally important, is the assessment of tinnitus, when treated with CI, by neuro-imaging using functional protocols. MRI imaging in CI patients gives rise to concerns regarding MRI-induced damage to the CI and artifacts caused by the implant [66,67]. Artifacts are induced by both the metallic parts of the CI and the magnet inside the implant. The magnet in the implanted part of the CI device provides a base for attachment of exterior speech processor and ensures wireless data transfer by means of inductive coupling. The induction of electrical current in metallic parts of the CI can cause magnetic field inhomogeneities and distort radiofrequency pulses. Although the first generation of CIs were incompatible with magnetic fields [68], recent generations of most CI brands are MRI compatible and hassle free up to 3 Tesla [69]. The artifacts caused by CIs in brain imaging remain a concern. In a study on cadaveric heads with previous types of non-removable magnets in 3 Tesla MRI, it was concluded that CI-induced artifacts show up as signal-void areas surrounding the implant are dependent on the sequence applied [66,67]. In addition to areas of signal void around the CI device, periodic shadowing obscures the whole brain in T2-weighted sequences. On T1-weighted images, anatomical structures on the implantation side are distorted, whereas structures on the contralateral side continue to be free of artifacts. In Figure 2, we show an example of a structural T1 scan of an individual with a CI implant compatible with a 3 Tesla MRI scanner, similar to another study [58]. The implant is banded on outside of the skin (so no real implantation) and clear artifacts can be observed.

**Figure 3:**
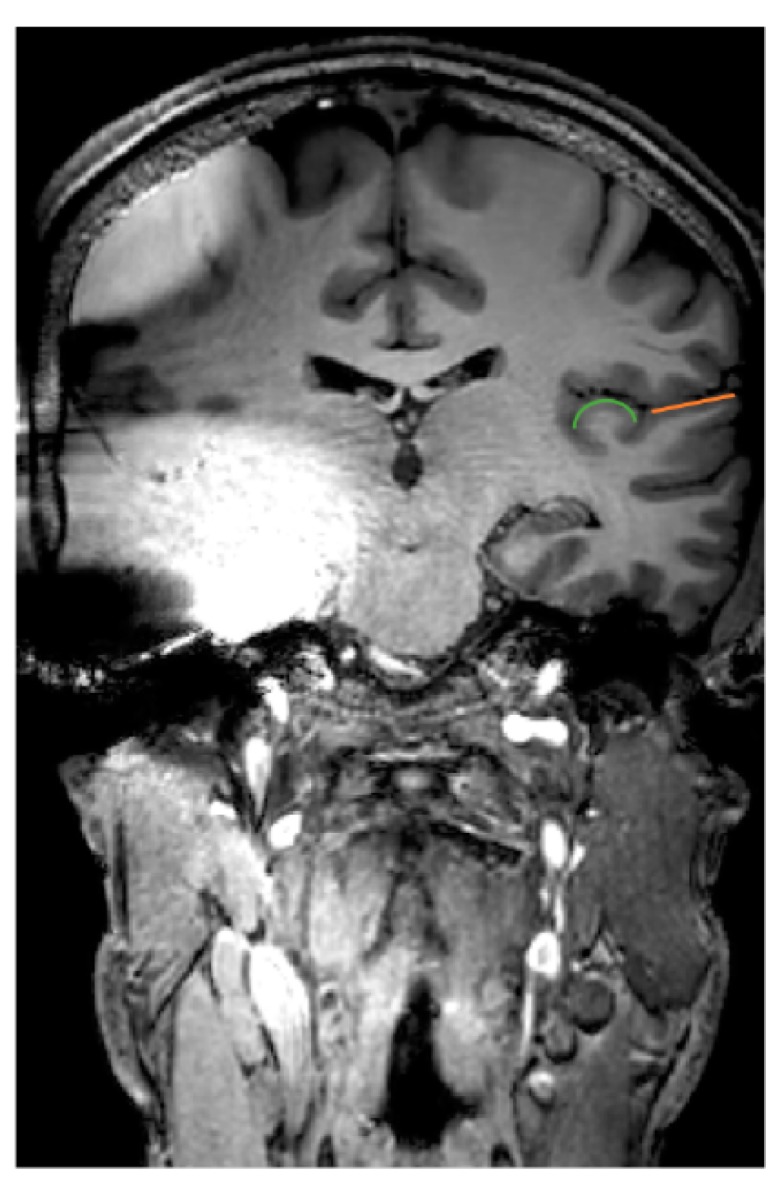
MRI artefacts caused by a unilateral CI on a coronal slice at the level of the auditory cortex. On the hemisphere contralateral to the device, the primary auditory cortex (Heschl’s gyrus) is depicted in *green* and the secondary auditory cortex (planum temporale) in *orange*.

In Figure 3, the impact on brain regions of interest is presented on a coronal slice. More specifically, on the contralateral hemisphere, the primary auditory cortex (i.e., Heschl’s gyrus) and the secondary auditory cortex (i.e., planum temporale) are depicted. These anatomical structures were impossible to be retrieved in the hemisphere ipsilateral to the CI device. This example could show that MRI scanning is possible without removal of the magnet or without the risk of dislocation. More extensive work with comparisons between the maximum implant magnet artefact size between 1.5 and 3 Tesla was performed in a recent study [70]. The authors concluded that no significant difference in artefact size was to be observed between several currently available CI systems.

## 5. Conclusions

In summary, the monaural hearing brain is a complex case, both with respect to processes underlying auditory deprivation and restoration. Research on the neural processes underlying unilateral auditory deprivation and restoration has expanded in the past decade. From a clinical perspective, evidence seems to favor early restoration of UHL to retain binaural hearing processes. However, the exact role of important factors such as the age of onset of UHL and the duration of UHL are to be further explored. Future research should be conducted to disambiguate these effects, ideally adopting longitudinal designs to investigate the neural plasticity of unilateral hearing deprivation. Moreover, as newer models of CIs become increasingly compatible and hassle-free with MRI scanners, tracing neural plasticity after restoration of the UHL will significantly increase our understanding, if metal artefacts are being tackled as well. Nevertheless, current insights seem to favor early restoration of the UHL, to avoid functional dominance of the hearing ear in the brain.

## Figures and Tables

**Table 1:** List of included articles on patients with unilateral hearing loss (UHL).

Year	Authors	Patients	Controls	Mean Age (Range)	Neural Method	Study Design	Duration of UHL
2000	Bilecen et al.	1 UHL	/	UHL: *M* = 53 y	fMRI	Case study	Pre- and post-UHL onset measures
2003	Khosla et al.	19 UHL	8 NH	UHL: *M* = 47 y (16–68 y) NH: *M* = 32 y (25–38 y)	EEG	Group	(1–4 y)
2005	Schmithorst et al.	4 R UHL, 4 L UHL	/	UHL: *M* = 9 ± 1.8 y	fMRI	Cohort	
2008	Lin et al.	12 UHL	10 NH	UHL: *M* = 30.8 ± 10.1 y NH: *M* = 31.1 ± 11.6 y	DW-MRI	Group	*M* = 15.4 y (1–48 y)
2009	Hanss et al.	18 UHL	16 NH	All: (27–59 y)	EEG	Group	L UHL: *M* = 6.6 ± 3.7 y R UHL: *M* = 5.5 ± 3.3 y
2009	Wu et al.	19 UHL	10 NH	UHL: *M* = 24.1 y (8–60 y) NH: *M* = 31 y (18–58 y)	DW-MRI	Group	(5– >20 y)
2010	Propst et al.	12 UHL	23 NH	UHL: *M* = 9.0 y (7.2–11.8 y) NH: *M* = 9.8 y (7.3–11.6 y)	fMRI	Group	
2011	Tibbetts et al.	16 UHL	10 NH	All: (7–17 y)	RS-fcMRI	Group	
2012	Burton, Firszt, et al.	26 UHL	24 NH	UHL: *M* = 47 y (25–72 y) NH: *M* = 47 y (25–71 y)	fMRI	Group	(0.2–72 y)
2013	Burton et al.	9 UHL	/	UHL: (28–53 y)	fMRI	Cohort	
2013	Clemmens et al.	128 UHL	/	UHL: *M* = 5.6 y (3 w–16 y)	T2 MRI	Cohort	
2013	Maslin, Munro, et al.	18 UHL	18 NH	UHL: *M* = 60 y (43–75 y) NH: *M* = 58 y (42–74 y)	EEG	Group	(6 m –7 y)
2013	Maslin, Munro, et al.	6 UHL	6 NH	UHL: *M* = 52 y (40–69 y) NH: *M* = 60 y (+/− 42–79 y)	EEG	Group	Measures 1-6 m post UHL
2014	Rachakonda et.al	179 HL	54 NH	All: (13–18 y)	DW-MRI	Group	Congenital and acquired
2014	Schmithorst et al.	21 UHL	23 NH	All: (7–12 y)	fMRI	Group	>2 years or unknown
2014	Wang et al.	17 L UHL, 17 R UHL	22 NH	L UHL: *M* = 45.7 ± 6.5 y R UHL: *M* = 43.0 ± 5.4 y NH: *M* = 46.0 ± 4.8 y	RS-fcMRI	Group	L UHL: *M* = 26.1 ± 10.9 m R UHL: *M* = 22.6 ± 11.7 m
2014	Yang, Chen, et al.	14 R UHL	19 NH	All: (41–60 y)	fMRI	Group	R UHL: *M* = 14.2 ± 14.9 y
2015	Liu et al.	19 UHL	35 NH	UHL: *M* = 48.6 ± 14.3 y NH: *M* = 53.2 ± 7.5 y	RS-fcMRI	Group	>1 y
2015	Pross et al.	8 L UHL, 4 R UHL	12 NH	UHL: *M* = 49 y (22–77 y) NH: *M* = 46 y (24–61 y)	MEG, MRI	Group	*M* = 10.4 y (2–25 y)
2015	Vos et al.	5 UHL	5 NH	UHL: *M* = 50.6 y (34–64 y) NH: *M* = 40.6 y (29–57 y)	DW-MRI	Group	(15–54 y)
2015	Zhang et al.	11 L UHL, 10 R UHL	11 NH	L UHL: *M* = 47.2 ± 10.8 y R UHL: *M* =55.2 ± 6.8 y NH: *M* = 51.7 ± 12.4 y	RS-fcMRI	Group	L UHL: *M* = 14.9 y (2–50 y) R UHL: *M* = 13.3 y (2–50 y)
2016	Van der Haegen	7 R UHL	7 NH	UHL: *M* = 45.0 y (29–70 y) NH: *M* = 45.3 y (31–70 y)	fMRI	Group	Congenital
2016	Zhang et al.	34 UHL	17 NH	UHL: *M* = 46.9 ± 14.6 y NH: *M* = 50.6 ± 13.5 y	RS-fcMRI	Group	L UHL: *M* = 13.8 ± 14.9 y R UHL: *M* = 17.3 ± 15.9 y
2017	Jung et al.	20 UHL	13 NH	All: (7–17 y)	RS-fcMRI	Group	
2017	Yamamoto et al.	5 L UHL, 7 R UHL	8 NH	UHL: *M* = 45.4 y (26–72 y) NH: *M* = 41.8 y (30–60 y)	fMRI	Group	*M* = 3.7 y (1–15 y)
2018a	Zhang et al.	17 L UHL, 21 R UHL	21 NH	L UHL: *M* = 46.6 ± 11.9 y R UHL: *M* = 50.1 ± 9.5 y NH: *M* = 43.8 ± 7.0 y	RS-fcMRI	Group	L UHL: *M* = 37.9 ± 14.1 m R UHL: *M* = 44.0 ± 13.6 m
2018b	Zhang et al.	17 L UHL, 21 R UHL	21 NH	L UHL: *M* = 46.6 ± 11.9 y R UHL: *M* = 50.1 ± 9.5 y NH: *M* = 43.8 ± 7.0 y	RS-fcMRI	Group	L UHL: *M* = 37.9 ± 14.1 m R UHL: *M* = 44.0 ± 13.6 m
2018c	Zhang et al.	21 UHL	21 NH	UHL: *M* = 44.2 ± 3.5 y NH: *M* = 42.8 ± 7.9 y	RS-fcMRI	Group	*M* = 34.0 ± 31.1 m
2019	Lipschitz et al.	189 UHL	/	UHL: (0–18 y)	MRI, CT	Cohort	Congenital and acquired

L = left, M = mean, NH = normal hearing, RS-fcMRI = resting state functional magnetic resonance imaging, DW-MRI = diffusion weighted MRI, fMRI = functional MRI, R = right, UHL = unilateral hearing loss, m = months, w = weeks, y = years old.

**Table 2:** List of included articles on monaural hearing restoration.

Year	Authors	Patients	Controls	Mean Age (Range)	Neural Method	Study Design	Duration of UHL
2013	Kral, Heid et al.	10 UHL cats	/	/	ABR	Animal study	Congenital
2013	Kral, Hubka et al.	7 UHL cats	7 NH cats	/	ABR	Animal study	Congenital
2013	Firszt et al.	1 UHL	/	UHL: 41 y		Case study	Probably congential
2015	Basta et al.	24 UHL guinea pigs	/	/	ABR	Animal study	Induced in adulthood
2016	Finke et al.	10 UHL	/	UHL: *M* = 53.2 y (26– 68 y)	ERP	Cohort study	*M* = 9.5 y (1-23 y)
2016	Sharma et al.	1 UHL	/	UHL: 9 years at CI implantation	CAEP	Case study	Progressive HL started at 5 y
2017	Canete et al.	1 UHL	/	UHL: 8 years at CI implantation	CAEP	Case study	Congenital
2017	Polonenko et al.	5 UHL	/	UHL: ≤ 3.6 y	EEG	Case study	Congenital
2019	Jakob et al.	22 UHL rats	19 NH rats	/	ABR	Animal study	Congenital

ABR = auditory brainstem response, CAEP = cortical auditory evoked potential, ERP = event-related potential, L = left, M = mean, NH = normal hearing, R = right, UHL = unilateral hearing loss, w = weeks, y = years old.
